# Role of mammographic templates in managing ever increasing workloads

**DOI:** 10.1186/bcr3796

**Published:** 2015-11-05

**Authors:** Anne Nielsen Moody, Maggie Fletcher, Nisha Sharma

**Affiliations:** 1 Leeds Teaching Hospitals NHS Trust, Leeds, UK

## Introduction

A breast imaging report is a key component of the breast cancer diagnostic process. The report must be clear and concise to avoid ambiguity and confusion. However, substantial variation in the information provided in a breast imaging report is not uncommon to see. We sought to develop a report template containing a summary of all essential information pertinent to the surgeons and the radiologists.

## Methods

Breast surgeons and radiologists were consulted as to what was required in a report and they stated breast density, correlation with clinical findings, lesion characteristics, R1−R5 category, site and size of lesion, and is clinical area of concern biopsied. A retrospective audit of 30 breast imaging reports of recently diagnosed carcinomas between October 2014 and January 2015 were reviewed to see if these were recorded.

## Results

Ten per cent of reports did not mention breast density. The most frequent information provided is lesion size (ultrasound 100 %, mammography 73 %). Correlation with referral was unclear in 10 %, R1−R5 category not given in 3 %. Site of lesion was not provided in 3 %. Seven per cent of the reports were 3−4 pages long, described as confusing and difficult to read by the two data extractors. Thirty per cent of reports were not separated into mammography/ultrasound/biopsy sections. There were 23 different ways of characterising lesions on mammography and 24 on ultrasound.

## Conclusion

The audit highlighted the need for a breast reporting template that met the needs of the clinicians to ensure the relevant facts were included to further improve the patient pathway.

